# Linking central sensitization to multisystemic manifestations in hypermobile Ehlers-Danlos syndrome

**DOI:** 10.3389/fpain.2026.1799439

**Published:** 2026-06-30

**Authors:** Ana Paula Montemayor Zarazúa, César Vidal Elizondo Solis, Camila Ayala García, Diego Jesús Pacheco Estrella, Octavio Ilizaliturri Guerra, Ana Cecilia Arana Guajardo, Emma Purón Gonzalez, Karina Silva Luna, Luis Iván Lozano Plata, Mario Ramon García Pompermayer, Mario Alberto Garza Elizondo

**Affiliations:** Insituto de Medicina Interna, Centro Médico Zambrano Hellion TecSalud, San Pedro Garza García, Nuevo León, México

**Keywords:** central sensitization, fatigue, hypermobility, multisystemic manifestations, pain

## Abstract

Hypermobile Ehlers-Danlos Syndrome (hEDS) and Hypermobility Spectrum Disorders (HSD) are complex multisystemic conditions frequently associated with chronic pain. Central Sensitization (CS)—a state of neural amplification and hyperexcitability—is hypothesized to be a unifying mechanism underlying the heterogeneous symptoms in chronic pain patients. Our aim was to investigate the association between central sensitization and multisystemic symptom burden in patients with hEDS/HSD while identifying independent clinical predictors of CS. We prospectively enrolled 150 adults diagnosed with hEDS/HSD at a specialized joint hypermobility clinic. Participants were evaluated using the Central Sensitization Inventory (CSI) and the SPIDER questionnaire. Clinical CS was defined as a CSI score > 40. Statistical analyses included univariate correlations and multivariable logistic regression. Centrally sensitized patients (*n* = 76) were significantly younger and predominantly female compared to the non-CS group. While CSI scores correlated strongly with all eight SPIDER domains (*p* < 0.001), a multivariable logistic regression model (AUC 0.98) identified only three independent predictors of CS: fatigue (OR 1.089), pain (OR 1.067), and cardiac dysautonomia (OR 1.057). Central sensitization in hEDS/HSD is independently associated with a triad of fatigue, pain, and cardiac dysautonomia. Clinical management should shift toward multidisciplinary strategies to effectively address the sensitized state in this population.

## 1 Introduction

Central Sensitization is a neuroplastic adaptation of the Central Nervous System (CNS) in response to repeated, prolonged, or particularly intense nociceptive input, such as chronic inflammation, recurrent injury, or stress, resulting in an increased sensitivity to stimuli (1). It can be defined as a functional, structural or chemical enhancement in neurons and circuits in nociceptive pathways that lowers their threshold of activation and amplifies their responses to received inputs, promoting the following: cell membrane excitability of central neurons; increased synaptic efficacy and strength; reduced transmission of inhibitory signals; and enlarged nociceptive-receptor fields. These neuroplastic changes in the CNS explain the pathophysiological occurrence of pain that sensitized patients experience in the presence of both noxious and innocuous stimuli (2, 3). Due to this heightened state of central hyperexcitability, the core clinical features of CS include hyperalgesia, allodynia, and global sensory hyperresponsiveness, in which patients exhibit augmented sensitivity to internal or external stimuli such as bright lights, odors, loud sounds, or visceral sensations including peristalsis. Beyond pain, CS may exert a multisystemic impact, commonly referred to as Central Sensitization Syndrome, which has been associated with fatigue, sleep disturbances, paresthesias, cognitive difficulties, irritable bowel syndrome, restless leg syndrome, urogenital symptoms, among others (4).

Hypermobile Ehlers–Danlos syndrome comprises a group of conditions primarily characterized by joint hypermobility and frequently accompanied by a wide range of multisystemic manifestations (5). This spectrum is commonly associated with generalized musculoskeletal pain of complex distribution, fatigue, and systemic symptoms that significantly impair patients' quality of life (6, 7). Increasing evidence suggests that CS contributes to both pain and multisystemic manifestations observed in hEDS/HSD, as highlighted by Di Stefano and colleagues, who described the heterogeneity of pathological pain in hEDS/HSD and proposed a predominant role of central sensitization in a subset of patients (8). With respect to pain in hEDS/HSD, it has also been characterized by clinical and experimental evidence following a three-phase progression in many patients; firstly acute, localized nociceptive pain from ligamentous/tendinous injury and joint instability, then emerging neuropathic features due to nerve compression and small-fiber alterations; and lastly sustained central sensitization resulting in generalized hyperalgesia and multisystemic symptoms. This staged model motivates the use of CSI scores and the SPIDER questionnaire, not only for pain itself but the other multisystemic features accompanying CS (9, 10).

The Central Sensitization Inventory is a validated self-report screening instrument that allows clinicians to assess, categorize, and quantify the severity of symptoms associated with CS (11). Similarly, the SPIDER questionnaire is a self-reported screening tool designed to identify symptoms related to CS, capturing not only pain intensity but also the multisystemic phenotype of centrally mediated pain (6). Together, these instruments provide complementary information for a comprehensive evaluation of pain and multisystemic symptom burden associated with central sensitization.

In this study, we sought to determine the association between central sensitization and multisystem manifestation in patients with hEDS/HSD. Characterizing these multisystemic connections is of particular importance, as clinical management of hEDS/HSD remains largely fragmented, frequently resulting in the compartmentalized treatment of overlapping systemic symptoms and, consequently, in reduced patient satisfaction. Disentangling the specific clinical domains that independently correlate with CNS hyperexcitability may not only guide future mechanistic investigations focused on targeted neurophysiological biomarkers, but also support the development of more integrated and effective multisystem phenotyping therapies for this patient population.

## 2 Materials and methods

### 2.1 Study design

This prospective observational study was conducted to characterize multisystemic symptom burden and central sensitization features in adults with hEDS/HSD. Patients were consecutively enrolled at the Joint Hypermobility Clinic of Centro Médico Zambrano Hellion between November 2024 and December 2025.

The study protocol was reviewed and approved by the Institutional Review Board and Ethics Committee of Centro Médico Zambrano Hellion (#05-2025). All procedures were conducted in accordance with the Declaration of Helsinki and its subsequent amendments. Participation was voluntary, and all participants provided written informed consent prior to enrollment.

### 2.2 Study population

A total of 150 adult patients with a clinical diagnosis of hEDS/HSD were enrolled. Inclusion criteria comprised age ≥18 years, a positive (>2 points) 5-Part Questionnaire indicative of generalized joint hypermobility, and a positive Beighton Score according to established thresholds (12, 13). All participants underwent a standardized clinical evaluation performed by a certified rheumatologist, including assessment of joint hypermobility using the Beighton Score and classification according to the 2017 hEDS/HSD diagnostic criteria (5). Participants unable to complete the study questionnaires or not meeting minimum diagnostic criteria were excluded.

### 2.3 Evaluations

All participants completed two validated, self-administered questionnaires. Multisystemic symptom burden was assessed using the SPIDER questionnaire, a 31-item Likert-scale instrument designed to quantify symptom severity across eight domains: neuromusculoskeletal, pain, cardiac dysautonomia, gastrointestinal, urogenital, anxiety, depression, and fatigue, with higher scores indicating greater symptom burden (6). Central sensitization was assessed using the Central Sensitization Inventory, which includes 25 symptom items in Section A and 10 items documenting prior diagnoses in Section B, yielding a total score ranging from 0 to 100. Clinically significant central sensitization was defined *a priori* as a CSI score greater than 40, in accordance with previously validated thresholds (14).

### 2.4 Data analysis

All statistical analyses were performed using Python and followed established best practices for clinical research. Descriptive statistics were used to summarize demographic and clinical characteristics. Continuous variables were evaluated for normality using visual inspection and the Shapiro–Wilk test and are presented as mean ± standard deviation or median with interquartile range, as appropriate, while categorical variables are summarized as frequencies and percentages.

Between-group comparisons for continuous variables were conducted using Student's t-test. Associations between total CSI scores and SPIDER domain scores were examined using Pearson's correlation coefficient, with correlation magnitudes interpreted using conventional effect size thresholds (15).

Multivariable logistic regression analyses were performed to evaluate the independent contribution of multisystem symptom domains to the presence of central sensitization, defined as a CSI score greater than 40. SPIDER domain scores were entered as predictors, and results are reported as odds ratios with corresponding 95% confidence intervals. Variance inflation factors were calculated to rule out collinearity. Statistical significance was defined as a two-tailed *p*-value < 0.05.

## 3 Results

### 3.1 Demographic and clinical characterization

The study population comprised 150 patients, stratified into a Central Sensitization group (*n* = 76) and a non-Central Sensitization group (*n* = 74) (Table 1). Patients in the CS group were significantly younger, with a median age of 36 years (IQR: 26.00–52.00) compared to 47 years (IQR: 30.25–57.75) in the non-CS group (*p* < 0.001). Biological sex distribution also differed significantly (*p* < 0.001), with the CS group being predominantly female (93.42%) compared to the non-CS group (70.27%). No significant differences were identified regarding hEDS/HSD subtypes (*p* = 0.344) or median Beighton scores (*p* = 0.052).

**Table 1:** Comparison of demographics, hypermobility spectrum subtype, and symptom severity between patients with or without central sensitization.

Variables	No CS (*n* = 74)	CS (*n* = 76)	*P*-value
Age, median (IQR)	47.00 (30.25–57.75)	36.00 (26.00–52.00)	<0.001
Sex			<0.001
Male, *n* (%)	22 (29.73%)	5 (6.58%)	
Female, *n* (%)	52 (70.27%)	71 (93.42%)	
Hypermobility spectrum			0.3445
L-HSD, *n* (%)	3 (4.05%)	4 (5.26%)	
G-HSD, *n* (%)	53 (71.62%)	55 (72.37%)	
H-HSD, *n* (%)	11 (14.86%)	6 (7.89%)	
P-HSD, *n* (%)	5 (6.76%)	5 (6.58%)	
hEDS, *n* (%)	0 (0.0%)	4 (5.26%)	
Beighton Score, median (IQR)	4.50 (3.00–6.00)	6.00 (4.00–7.00)	0.05236
SPIDER			
NMSK, median (IQR)	15.00 (5.00–25.00)	40.00 (30.00–50.00)	<0.001
Pain, median (IQR)	18.75 (12.50–25.00)	43.75 (31.25–57.81)	<0.001
Fatigue, median (IQR)	16.67 (8.33–25.00)	58.33 (50.00–75.00)	<0.001
Cardiac Dysautonomia, median (IQR)	5.00 (0.00–18.44)	35.63 (15.94–54.06)	<0.001
GI, median (IQR)	12.50 (0.00–18.75)	37.50 (25.00–56.25)	<0.001
UG, median (IQR)	6.66 (0.00–11.66)	20.00 (10.00–35.00)	<0.001
Anxiety, median (IQR)	16.67 (8.33–31.25)	50.00 (33.33–68.75)	<0.001
Depression, median (IQR)	8.33 (0.00–16.67)	41.67 (16.67–58.33)	<0.001

IQR, interquartile range; L-HSD, localized hypermobility spectrum disorder; G-HSD, generalized hypermobility spectrum disorder; H-HSD, historical hypermobility spectrum disorder; P-HSD peripheral hypermobility spectrum disorder; hEDS, Hypermobile Ehlers Danlos Syndrome.

Regarding clinical severity, the CS group demonstrated significantly higher median scores across all SPIDER domains (all *p* < 0.001). Notably, the CS group reported markedly higher scores for fatigue (58.33 vs. 16.67), pain (43.75 vs. 18.75), and cardiac dysautonomia (35.63 vs. 5.00). Significant elevations in the CS group were also noted for gastrointestinal (37.50 vs. 12.50) and urogenital (20.00 vs. 6.66) symptoms, alongside substantially higher scores for anxiety (50.00 vs. 16.67) and depression (41.67 vs. 8.33).

### 3.2 Multisystemic correlations with central sensitization

**Figure 1:**
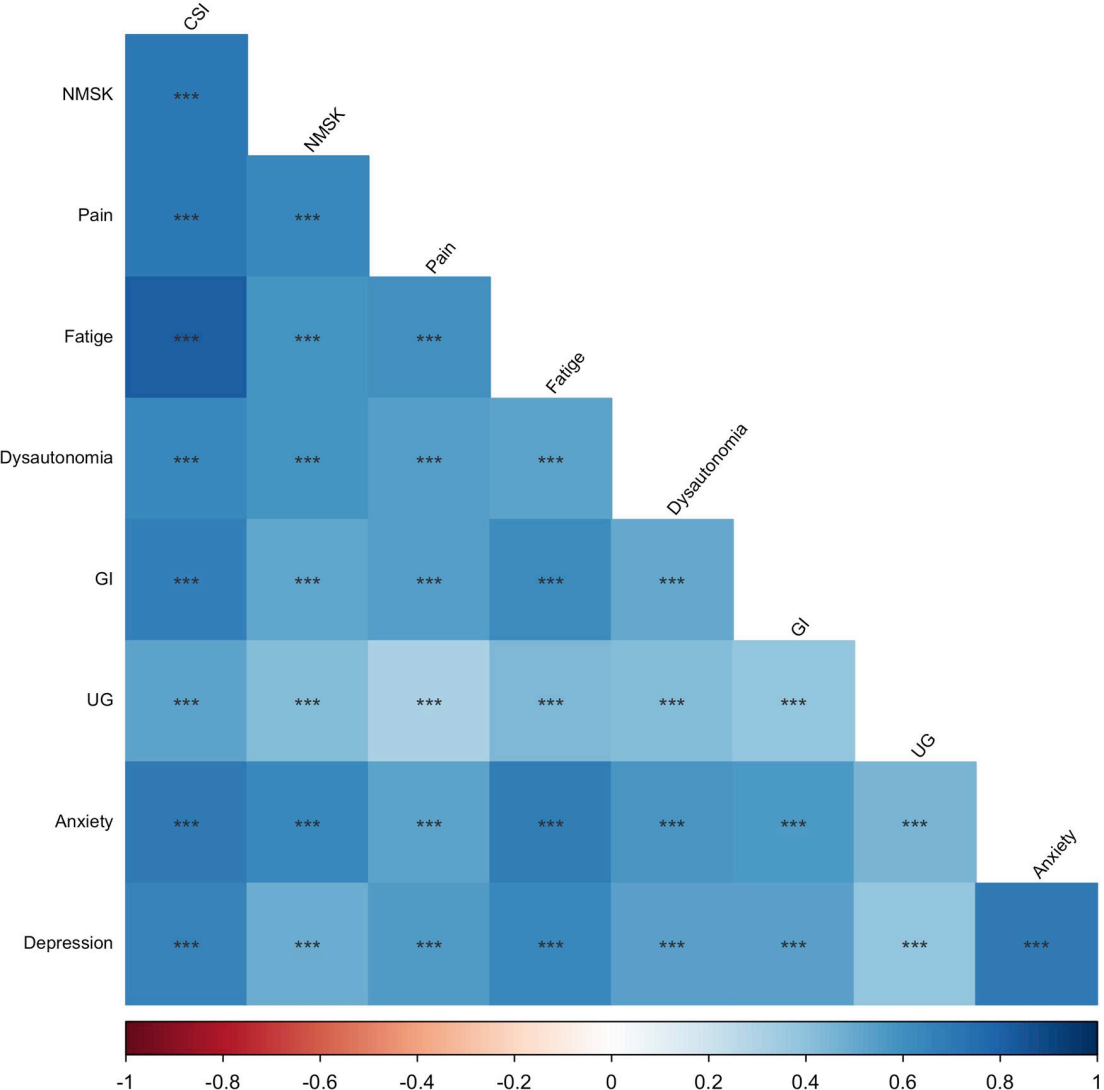
Central sensitization in hEDS/HSD patients highly correlates with multisystemic impact. The correlation matrix shows significant positive associations across all measured domains, including CSI, pain, fatigue, dysautonomia, GI and UG symptoms, anxiety, and depression. Color intensity reflects the strength of the positive correlation. *** indicates significance at *p* < 0.001.

Univariate correlation analysis revealed robust, significant positive associations (all *p* < 0.001) between CSI scores and all multisystemic domains measured by the SPIDER questionnaire (Figure 1). The strongest associations were observed for fatigue (r = 0.71), pain (r = 0.70), and cardiac dysautonomia (r = 0.67). High inter-domain correlations were also identified, particularly between anxiety and depression (r = 0.81) and between anxiety and fatigue (r = 0.68).

### 3.3 Multivariate analysis of central sensitization determinants

To identify the independent determinants of central sensitization —defined as a CSI score greater than 40 points—we performed a comprehensive multivariable logistic regression analysis incorporating all eight clinical domains of the SPIDER questionnaire simultaneously. This simultaneous adjustment was specifically designed to address potential construct overlap between the two instruments, and subsequent collinearity diagnostics confirmed that the model parameters were highly stable, with all Variance Inflation Factors (VIF) for the individual domains remaining well below conservative thresholds, ranging from 1.46 to 3.31. Ultimately, the model demonstrated excellent predictive performance, yielding an Area Under the Receiver Operating Characteristic curve (AUC) of 0.98 (95% CI: 0.95–1.00) and a Tjur's R2 of 0.78. This robust statistical fit translated into an overall classification accuracy of 93.3%, characterized by a highly balanced positive (93.4%) and negative (93.2%) predictive power.

Despite the widespread correlations observed in the univariate analysis, only three domains emerged as significant independent predictors of central sensitization: fatigue, pain, and cardiac dysautonomia (Table 2). Fatigue remained the strongest predictor in the model (OR: 1.089; 95% CI: 1.049–1.142). Similarly, higher scores in Pain (OR: 1.067; 95% CI: 1.020–1.129) and cardiac dysautonomia (OR: 1.057; 95% CI: 1.013–1.111) were significantly associated with an increased likelihood of central sensitization. Conversely, the domains of anxiety, depression, gastrointestinal, urogenital, and neuromusculoskeletal symptoms did not show a statistically significant association with CS positivity in this multivariate context.

**Table 2:** Multivariable logistic regression analysis of SPIDER domains as predictors of CSI positivity.

Spider Domain	*β*	SE	OR	CI
Fatigue	0.085	0.021	1.089	[1.049, 1.142]
Pain	0.065	0.026	1.067	[1.020, 1.129]
Cardiac Dysautonomia	0.055	0.023	1.057	[1.013, 1.111]
NMSK	0.025	0.026	1.025	[0.975, 1.081]
Gastrointestinal	0.023	0.021	1.024	[0.983, 1.069]
Urogenital	0.003	0.022	1.003	[0.960, 1.050]
Anxiety	−0.001	0.021	0.999	[0.958, 1.043]
Depression	−0.001	0.019	0.999	[0.961, 1.035]
Constant	−7.497	1.364	—	—

β, beta regression coefficient; SE, standard error; OR, Odds Ratio; CI, 95% confidence interval.

## 4 Discussion

This study demonstrates a robust and consistent association between central sensitization and the plethora of independent multisystem manifestations in patients with hEDS/HSD. While previous literature has established CS as a mechanism for pain amplification, our results extend this concept, showing that higher CSI scores correlate significantly with increased severity across all multisystemic domains assessed by the SPIDER questionnaire. This reinforces the hypothesis that a state of central hyperexcitability is highly associated with the heterogeneous symptoms reported by the hEDS/HSD population (16). Our findings align with recent models suggesting that generalized hyperalgesia in hypermobility evolves through distinct phases, beginning with localized nociceptive triggers and progressing to a generalized central state characterized by heightened sensory responsiveness and dysregulated autonomic processing (9).

A critical finding from our multivariate analysis is the identification of a specific triad pertinent to central sensitization: fatigue, pain, and cardiac dysautonomia. The emergence of fatigue as the strongest independent predictor (OR 1.089) is particularly significant. Chronic fatigue in hEDS/HSD may reflect a centralized neurobiological alteration, where continuous stimulation of peripheral nociceptors triggered by mediators released from an aberrant extracellular matrix, leads to central neuroinflammation (17, 18). This “bottom-up” signaling from affected ligaments, tendons, and connective tissue creates a persistent nociceptive barrage that maintains the sensitized state. Furthermore, the independent contribution of cardiac dysautonomia suggests that sympathetic overactivation may lower the threshold for sensory processing, effectively “priming” the central nervous system for hypersensitivity (3, 19).

The strong predictive value of pain in our model must be viewed through the lens of the complex “pain phenotyping” seen in Ehlers-Danlos subtypes (20). In hEDS/HSD, pain is often a hybrid of nociceptive, neuropathic, and nociplastic components. Early-stage nociceptive pain stems from joint instability, microtrauma, and ligamentous damage. However, as noted in recent literature, a significant neuropathic component—evidenced by decreased intraepidermal nerve fiber density and axonal neuropathies—often coexists (18). Moreover, the loss of proprioception in these patients likely exacerbates this cycle; impaired balance and coordination lead to joint overload and disuse deconditioning, which in turn increases functional disability and provides the persistent peripheral input necessary to sustain central sensitization (20).

Our results offer a crucial nuance regarding the role of psychological factors. Although Anxiety and Depression showed strong univariate correlations with CSI scores, they did not retain significance in the multivariate model. This suggests that while anxiety and depression may intensify the pain experience, they likely function as secondary manifestations of the profound physical and autonomic burden (21). This challenges the psychosomatic stigma often faced by this population and supports a more somatic-focused phenotyping. Furthermore, the observation that the CS group was significantly younger (median 36 years) suggests that sensitization may be more aggressive in earlier stages of the disease, perhaps related the acute phase of joint instability before compensatory mechanisms or “stiffening” occur with age (22).

From a clinical perspective, these results highlight the necessity of moving toward a comprehensive “phenotyping” of hEDS/HSD pain. Recognizing that sensitization is independently associated with a triad of fatigue, pain, and cardiac dysautonomia suggests that therapeutic approaches must extend beyond simple analgesia. Interventions should include autonomic stabilization, proprioceptive training to reduce nociceptive “noise,” and metabolic support for fatigue. Our data supports the use of validated tools like the CSI and SPIDER to reduce diagnostic fragmentation. Moreover, beyond questionnaire-based assessment, objective sensory phenotyping offers an opportunity to further refine pain characterization in hypermobility-related disorders. Sensory profiling studies in classical Ehlers-Danlos syndrome have demonstrated vibration hypoesthesia, altered thermal sensation, mechanical hyperalgesia, and impaired pain modulation (16). Applying similar methodologies in hEDS/HSD could help identify distinct pain phenotypes, such as small-fiber predominant vs. central-gain-predominant profiles. Until such data is available, comprehensive therapeutic approaches that address central mechanisms, alongside peripheral and biomechanical factors, may therefore be essential to optimizing symptom management and improving quality of life in this complex patient population. Future research should evaluate whether targeted interventions aimed at these central mechanisms—rather than focusing on isolated psychological or peripheral symptoms—can modify the disease trajectory and improve functional outcomes across different age groups.

Despite the hefty predictive power of the model, several limitations must be acknowledged. First, its cross-sectional design prevents establishing definitive causal relationships, necessitating longitudinal studies to determine if these independent predictors precede or result from central sensitization. Notably, this study was specifically designed as an intra-cohort phenotypic analysis of the hEDS/HSD spectrum rather than a comparative trial against healthy or non-hypermobile chronic pain populations, the absence of an external control group means the identified fatigue, pain, and cardiac dysautonomia triad cannot be declared pathognomonic or unique to this spectrum; nonetheless, characterizing these internal relationships remains critical. Second, the sample size, while sufficient for the current multivariate analysis, may limit the generalizability of the findings to all hEDS/HSD phenotypes. Third, the reliance on self-reported clinical screening questionnaires lacks objective sensory or neurophysiological assessment, such as QST, CPM or objective autonomic reflex testing. Consequently, our interpretation regarding underlying central and autonomic nervous system mechanisms remain theoretical. Fourth, our cohort was recruited from a specialized tertiary joint hypermobility clinic, which inherently introduces a selection bias by enriching the sample with patients experiencing greater multisystemic symptom severity. Therefore, these findings may not fully generalize to milder, asymptomatic individuals in the hypermobility spectrum, community-based cohorts or primary care settings. Finally, the significant demographic imbalance regarding sex and the younger age of sensitized patients may reflect a specific clinical subpopulation, necessitating further research to validate these findings in male patients and older cohorts.

## 5 Conclusion

In conclusion, this study demonstrates that while central sensitization in hEDS/HSD involves a broad multisystemic impact, it is independently associated with a triad of fatigue, pain, and cardiac dysautonomia. These results underscore the need for a clinical shift toward integrated, multi-system phenotyping therapies, over fragmented, symptom-specific treatments. By targeting these fundamental central mechanisms, clinicians might appease global symptom burden and improve quality of life in this complex population.

## Data Availability

The original contributions presented in the study are included in the article/Supplementary Material, further inquiries can be directed to the corresponding authors.
